# NAMPT/SIRT1 Attenuate Ang II-Induced Vascular Remodeling and Vulnerability to Hypertension by Inhibiting the ROS/MAPK Pathway

**DOI:** 10.1155/2020/1974265

**Published:** 2020-12-30

**Authors:** Lei Zhou, Sheng Zhang, Enkhbat Bolor-Erdene, Lingwei Wang, Ding Tian, Yunqing Mei

**Affiliations:** ^1^Department of Cardiothoracic Surgery, Tongji Hospital, School of Medicine, Tongji University, Shanghai 200065, China; ^2^Department of Cardiology, East Hospital, School of Medicine, Tongji University, 310000, China; ^3^Department of Thoracic Surgery, East Hospital, School of Medicine, Tongji University, Shanghai 310000, China; ^4^Department of Cardiology, Xinhua Hospital, School of Medicine, Shanghai Jiao Tong University, Shanghai 200092, China

## Abstract

Hypertension is characterized by endothelial dysfunction, vascular remodeling, and rearrangement of the extracellular matrix. Besides, the pathogenesis of hypertension is closely related to excess generation of reactive oxygen species (ROS). Nicotinamide phosphoribosyltransferase (NAMPT) is a rate-limiting enzyme in nicotinamide adenine dinucleotide (NAD) biosynthesis that influences the activity of NAD-dependent enzymes, such as sirtuins, which possess NAD-dependent protein deacetylase activity and cleave NAD during the deacetylation cycle. Recently, NAMPT has been shown to play a crucial role in various diseases associated with oxidative stress. However, the function and regulation of NAMPT in hypertension have not been extensively explored. In the present study, we identified NAMPT as a crucial regulator of hypertension, because NAMPT expression was significantly downregulated in both patients with hypertension and experimental animals. NAMPT knockout (NAMPT+/-) mice exhibited a significantly higher blood pressure and ROS levels after stimulation with angiotensin II (Ang II) than wild-type mice, and the administration of recombinant human NAMPT (rhNAMPT) reversed this effect. In vivo, overexpression of NAMPT protected against angiotensin II- (Ang II-) induced hypertension by inhibiting ROS production via sirtuin 1 in mouse aortic endothelial cells (MAECs) and mouse aortic vascular smooth muscle cells (MOVAs). In turn, NAMPT alleviated the ROS-induced mitogen-activated protein kinase (MAPK) pathway. In conclusion, NAMPT might be a novel biomarker and a therapeutic target in hypertension.

## 1. Introduction

As an important and independent risk factor for cardiovascular disease (CVD), hypertension affects 30% of adults and has been the leading risk factor for heart attack and stroke [1]. Angiotensin II (Ang II), the initial effector molecule of the renin-angiotensin system (RAS), has been shown to induce NAD(P)H oxidase (NOX) activity and increase local reactive oxygen species (ROS) production by binding to AT1R [2]. ROS are novel intracellular second messengers in signal transduction pathways. ROS-induced endothelial dysfunction appears to be the key early step in the development of hypertension [3]. The stimulation of vascular smooth muscle cell (VSMC) proliferation and rearrangement of extracellular matrix (ECM) formation are the major adaptive mechanisms involved in vascular alterations [4]. The ROS-sensitive mitogen-activated protein kinase (MAPK) cascade is also presumed to play a crucial role in endothelial cell (EC) dysfunction, the proliferation and migration of VSMCs, and formation of ECM through Ang II and ROS [5, 6].

Nicotinamide phosphoribosyltransferase (NAMPT) is one of the regulators of the intracellular nicotinamide adenine dinucleotide (NAD) pool through its NAD biosynthesis activity [8]. NAMPT influences the activity of NAD-dependent enzymes, such as sirtuins [7], poly(ADP-ribose) polymerases (PARPs) [9], and CD38 [10], thereby regulating cellular metabolism, mitochondrial biogenesis, and adaptive responses to inflammation and oxidation. NAMPT is expressed in nearly all examined organs, tissues and cells [11] and has dual intracellular (mainly in the cytoplasm and nucleus) and extracellular roles [12]. NAMPT has been reported to be involved in various diseases and conditions, such as obesity, nonalcoholic fatty liver disease (NAFLD), type 2 diabetes mellitus (T2DM), cancer, and aging [11]. In the cardiovascular system, NAMPT promotes atherosclerosis development by exacerbating atherosclerotic inflammation [13]. NAMPT inhibits myocardial injury by regulating NAD and ATP levels [14]. NAMPT was recently suggested to play a role in pulmonary vascular remodeling, and NAMPT inhibition might be a potential therapeutic strategy for pulmonary arterial hypertension [15]. However, up to date, the molecular mechanism of NAMPT and its function in hypertension associated with oxidative stress have not been well studied.

In the present study, we identified NAMPT as a crucial regulator of hypertension, because the expression of NAMPT was significantly downregulated in both patients with hypertension and experimental animals. NAMPT knockout (NAMPT+/-) mice displayed significantly higher blood pressure and ROS levels than wild-type (WT) mouse littermates. Moreover, the administration of recombinant human NAMPT (rhNAMPT) reversed this effect in vivo. Overexpression of NAMPT protected against Ang II-induced increases in ROS levels through sirtuin 1 (SIRT1) and NAD+. In turn, NAMPT alleviated the ROS-induced MAPK pathway. These results confirm that approaches targeting or using NAMPT as a therapeutic option are potential strategies for the treatment of hypertension.

## 2. Results

### 2.1. NAMPT Expression Is Reduced in Tissues from Individuals with Hypertension, Mouse Aortic Vascular Smooth Muscle Cells (MOVAs), and Mouse Aortic Endothelial Cells (MAECs) Exposed to Ang II

**Figure 1:**
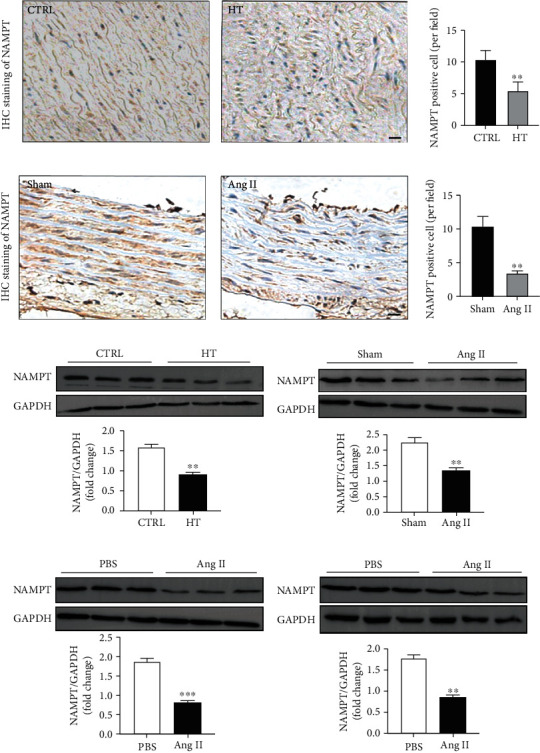
NAMPT expression is reduced in tissues from patients with hypertension and MOVAs and MAECs exposed to Ang II. (a) Immunohistochemical staining for NAMPT in tissues from patients with hypertension and control patients (scale bar = 20 *μ*m) and quantification of NAMPT-positive cells (*n* = 6 in each group, ≥40 fields per group). Data are presented as means ± SEM. ^∗∗^*p* < 0.01 compared with the group. (b) Immunohistochemical staining for NAMPT in tissues from the Ang II-infused rats and sham rats (scale bar = 20 *μ*m) and quantification of NAMPT-positive cells (*n* = 6 in each group, ≥40 fields per group). Data are presented as means ± SEM. ^∗∗^*p* < 0.01 compared with the group. (c) Representative Western blots and quantification showing NAMPT levels in the patients with hypertension and control patients (*n* = 6 in each group). (d) Representative Western blots and quantification showing NAMPT levels in the Ang II-infused rats and sham rats (*n* = 6 in each group). Representative Western blots and quantification showing NAMPT levels in MAECs (e) and MOVAs (f) after Ang II stimulation for 48 hours. Data are presented as means ± SEM and ^∗∗∗^*p* < 0.001 compared with the sham or PBS groups.

Aortic wall samples were obtained from patients undergoing aortic replacement surgery due to acute aortic dissection to explore whether NAMPT is involved in the development of hypertension. The clinical characteristics of patients with hypertension and normal controls undergoing aortic replacement are shown in Table 1. Immunochemical staining and Western blotting revealed a slight decrease in NAMPT levels in patients with hypertension (Figures 1(a) and 1(c)). Ang II-induced hypertensive rats were used as a hypertension model in this study. The rats were continually stimulated with Ang II through osmotic minipumps for 4 weeks, and fresh aortic wall tissues were collected; immunochemical staining and Western blotting revealed a significant reduction in Ang II-induced hypertension in rats (Figures 1(b) and 1(d)). MOVAs and MAECs were treated with Ang II for 48 hours to further investigate the role of NAMPT in vitro. The Western blot analysis revealed a marked decrease in the level of the NAMPT protein in both of these cell lines after exposure to Ang II (Figures 1(e) and 1(f)).

### 2.2. NAMPT Haplodeficiency Exacerbates Increases in Blood Pressure and Promotes the Expression of Remodeling-Related Molecules after Ang II Stimulation

**Figure 2:**
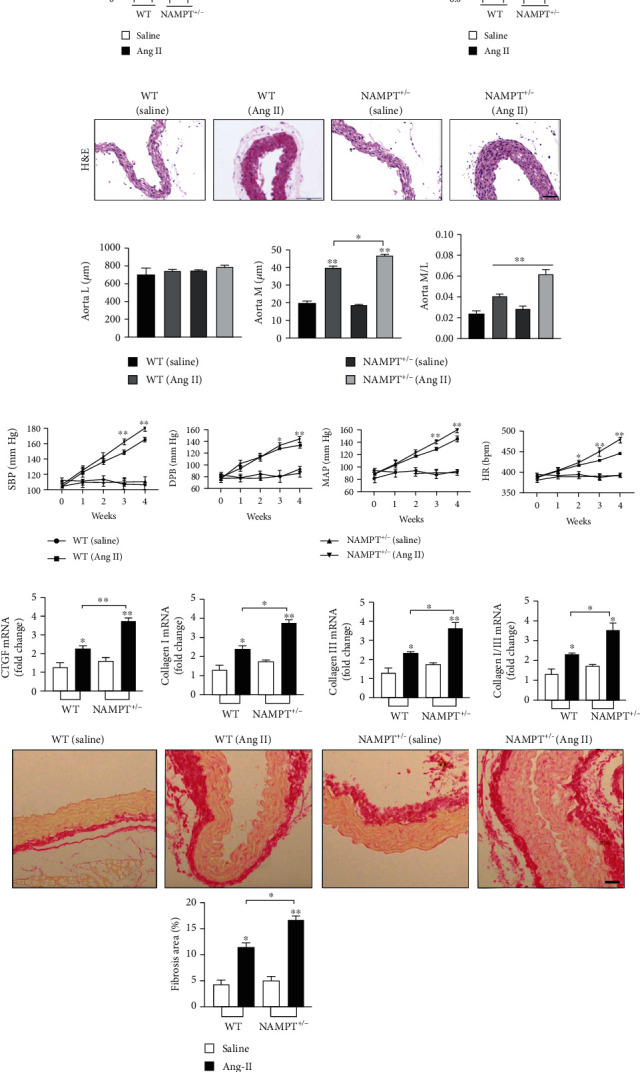
NAMPT haplodeficiency exacerbates changes in blood pressure and promotes the expression of remodeling-related molecules after Ang II stimulation. (a) The levels of the NAMPT mRNA and protein in the aortic wall tissues obtained from WT and NAMPT+/- mice treated with saline or Ang II (*n* = 6 in each group). (b) Representative images of H&E staining showing the remodeling of arteries (scale bar = 50 *μ*m, *n* = 5 in each group, ≥40 fields per group). (c) M, L, and their ratio in the aorta. (d) The SBP, MAP, DBP, and HR were measured with a noninvasive computerized tail-cuff system in conscious mice. (e) Relative mRNA levels of CTGF, collagen I, and collagen III and the ratio of collagen type I and type III in WT littermates and NAMPT+/- mice treated with saline or Ang II treatment (*n* = 6 animals per group), as well as the picrosirius red staining (PSR). Data are presented as means ± SEM. ^∗^*p* < 0.05 and ^∗∗^*p* < 0.01 compared with the corresponding saline group; NS: not significant.

We generated NAMPT haplodeficient (NAMPT+/-) mice to determine the effects of NAMPT on Ang II-induced hypertension. 32.16% decrease in the expression of the NAMPT mRNA and a 30.11% decrease in the level of the NAMPT protein were observed in the aortic walls of NAMPT+/- mice compared to their WT littermates. Moreover, a 40.46% decrease in the expression of the NAMPT mRNA and a 50.37% decrease in the aortic walls of NAMPT+/- mice were observed after Ang II stimulation (Figures 2(a) and 2(b)). Hematoxylin and eosin (H&E) staining was performed to further analyze morphological changes in mice with Ang II-induced hypertension (Figure 2(b)). We determined the media thickness (M), lumen diameter (L), and the ratio of media thickness to lumen diameter (M/L) in the different groups of mice to evaluate the remodeling of the aortic wall. Compared with control mice, Ang II-stimulated NAMPT+/- mice displayed significantly increased aortic M and M/L. However, statistically significant changes in L were not observed between groups (Figure 2(d)). Then, we measured the blood pressure of NAMPT+/- mice and age-matched control mice after Ang II stimulation for 28 days. The NAMPT+/- mice showed a slight increase in systolic blood pressure (SBP), mean blood pressure (MBP), and diastolic blood pressure (DBP) beginning at the third week (Figure 2(e)). A detailed description of the blood pressure parameters is provided in Table S2. We examined the mRNA levels of remodeling-related molecules in the aortic wall to further determine the pathological consequences. The qRT-PCR analysis revealed the downregulation of the Ang II-induced expression of remodeling markers, including connective tissue growth factor (CTGF), collagen I, and collagen III, in NAMPT+/- mice compared to WT mice after Ang II stimulation (Figure 2(f)), as well as the picrosirius red staining (PSR). Based on these results, NAMPT haplodeficiency significantly exacerbates Ang II-induced hypertension.

### 2.3. Ang II-Induced Endothelial Dysfunction and Proliferation and Migration of MOVAs Were Ameliorated by the Overexpression of NAMPT

**Figure 3:**
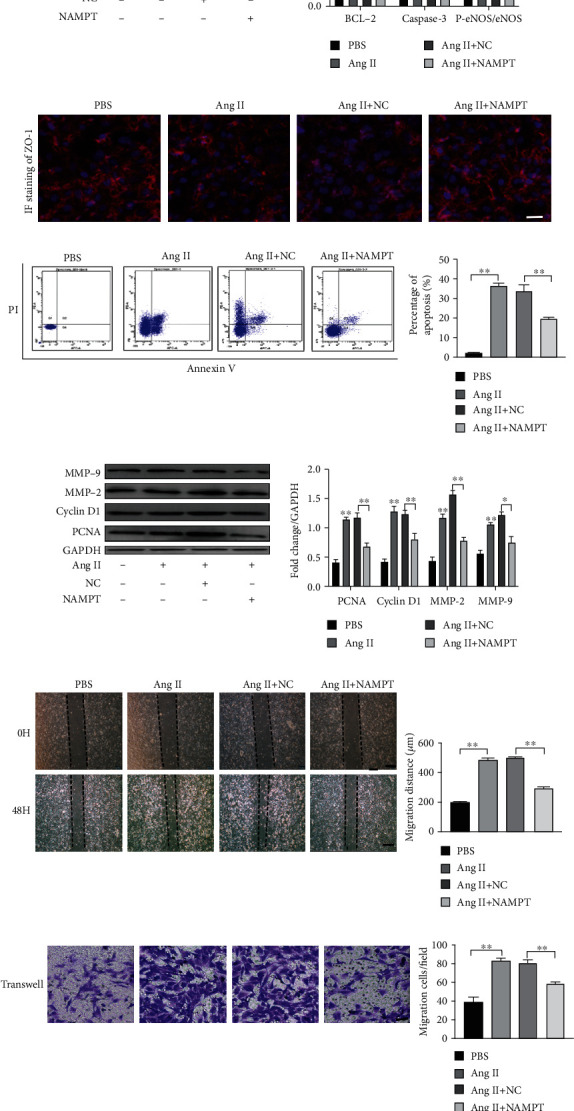
Ang II-induced endothelial dysfunction and changes in the proliferation and migration of MOVAs are ameliorated by the overexpression of NAMPT. (a) The protein levels and densitometric quantification of apoptosis markers (Caspase 3 and Bcl-2) and P-eNOS/eNOS were determined in cells transfected with the NAMPT plasmid and treated with Ang II for 48 h using Western blot analyses. (b) The expression of the endothelial cell permeability marker ZO-1 in MAECs was determined using immunofluorescence staining (ZO-1: red; nuclei (DAPI): blue). Scale bar = 500 *μ*m. (c) MAECs subjected to different treatments were stained with Annexin V and propidium iodide (PI) and analyzed using flow cytometry to evaluate the effect of NAMPT on cell apoptosis after the Ang II treatment. Quantification of cell apoptosis is showed in the right panel. (d) Relative levels of proliferation-related proteins (PCNA and cyclin D1) and extracellular matrix synthesis-related proteins (MMP-2 and MMP-9) in cultured MOVAs from the indicated groups. (e, f). Representative images of scratch-wound assays and transwell assays and quantification of migrated MOVAs (*n* = 4 in each group; scale bar = 200 *μ*m). Data are presented as means ± SEM.^∗^*p* < 0.05, ^∗∗^*p* < 0.01, and ^∗∗∗^*p* < 0.001 compared with the Ctrl or between the two indicated groups.

NAMPT was overexpressed through plasmid transfection, and cells were stimulated with Ang II for 48 hours to investigate the precise biological function of NAMPT in regulating Ang II-induced endothelial dysfunction. NAMPT expression in MAECs was substantially upregulated at 48 hours after transfection with the NAMPT plasmids, as measured by qRT-PCR and Western blotting (Fig. S1A and B). Moreover, the protein levels of apoptosis markers (Caspase 3 and Bcl-2) were significantly reduced in NAMPT-overexpressing cells stimulated with Ang II (Figure 3(a)), which was also assessed using double PI/Annexin V staining and flow cytometry (Figure 3(c)). Similar results were obtained at the level of endothelial nitric oxide synthase (P-eNOS/eNOS) (Figure 3(a)). Next, immunofluorescence staining further clarified that the dysregulation of ZO-1 protein (an EC cell permeability marker) was rescued by overexpression of NAMPT (Figure 3(b)). The transfection efficiency of NAMPT plasmids was determined using qRT-PCR and Western blotting (Fig. S1C and D. The increased protein levels of proliferation markers (proliferating cell nuclear antigen (PCNA) and cyclin D1) after stimulation were significantly blunted by the overexpression of NAMPT in MOVAs (Figure 3(d)). Moreover, in vitro scratch-wound assays and transwell migration assays revealed that Ang II-induced increases in cellular migration were significantly alleviated by NAMPT overexpression in MOVAs (Figures 3(e) and 3(f)). To further determine the role of NAMPT in ECM synthesis, levels of the fibronectin and matrix metalloproteinase (MMP-2 and MMP-9) were evaluated and were significantly reduced in Ang II-stimulated MOVAs that were transfected with the NAMPT plasmid (Figure 3(d)).

### 2.4. NAMPT Regulated SIRT1 Expression and the NAD Concentration In Vivo and In Vitro

**Figure 4:**
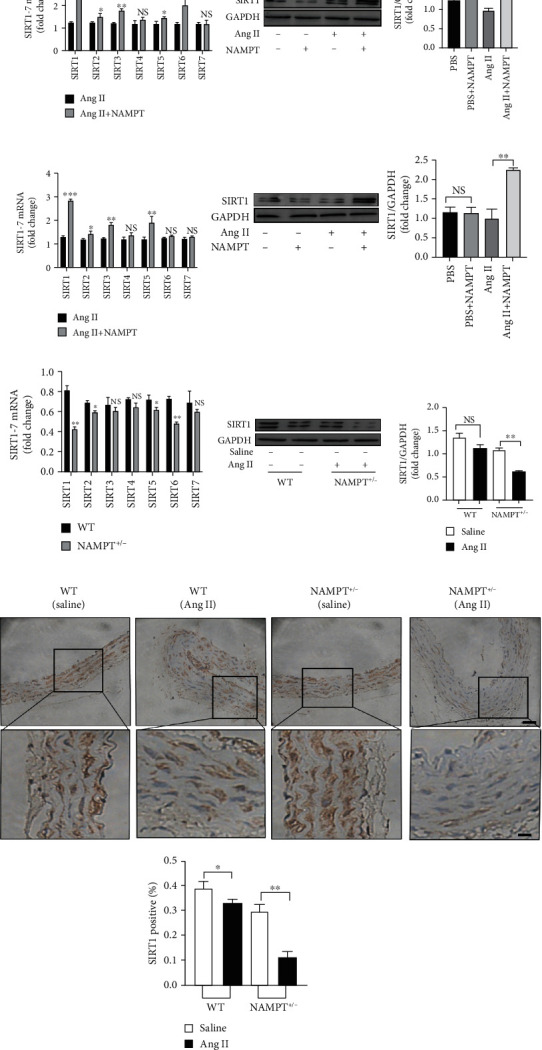
NAMPT regulates SIRT1 expression in vivo and in vitro. Representative qRT-PCR results and summarized data showing the expression of SIRT1-7 in MAECs (a) and MOVAs (c) transfected with NAMPT and stimulated with Ang II. Representative Western blot and summarized data showing the levels of SIRT1 in MAECs (b) and MOVAs (d) transfected with NAMPT plasmids and stimulated with Ang II. (e) Representative qRT-PCR and summarized data showing the expression of SIRT1-7 from NAMPT haplodeficient (NAMPT+/-) mice and their littermate control mice after Ang II stimulation (*n* = 4 in each group). (f) Representative Western blot and quantitative analysis of SIRT-1 levels among the different groups. (g) Immunohistochemical staining showing the level of SIRT1 in Ang II-treated NAMPT haplodeficient (NAMPT+/-) mice and their littermate control mice (*n* = 4 in each group; scale bar 50 *μ*m). Data are presented as means ± SEM. ^∗^*p* < 0.05, ^∗∗^*p* < 0.01, and ^∗∗∗^*p* < 0.001, and NS indicates no significant difference between the 2 indicated groups.

Because NAMPT mainly regulates the SIRT family in the cardiovascular system, we used qRT-PCR to examine SIRT1-7 expression in MAECs and MOVAs transfected with the NAMPT plasmid and stimulated with Ang II to determine which SIRTs play the most important role in the development of hypertension. Interestingly, we found the SIRT1 mRNA was expressed at higher levels both in MAECs (Figure 4(a)) and MOVAs (Figure 4(c)) than the other SIRT family members. Next, Western blot analysis was performed to verify the level of the Sirt1 protein and showed increased levels in both MAECs (Figure 4(b)) and MOVAs (Figure 4(d)) transfected with the NAMPT plasmid and stimulated with Ang II. Immunohistochemical staining further confirmed a robust decrease in the level of SIRT1 in the aortic walls of Ang II-induced NAMPT+/- mice and control mice (Figure 4(e)).

### 2.5. NAMPT Alleviated Ang II-Induced Endothelial Injury and the Proliferation and Migration in MOVAs by Targeting NAD/SIRT1 Signaling

**Figure 5:**
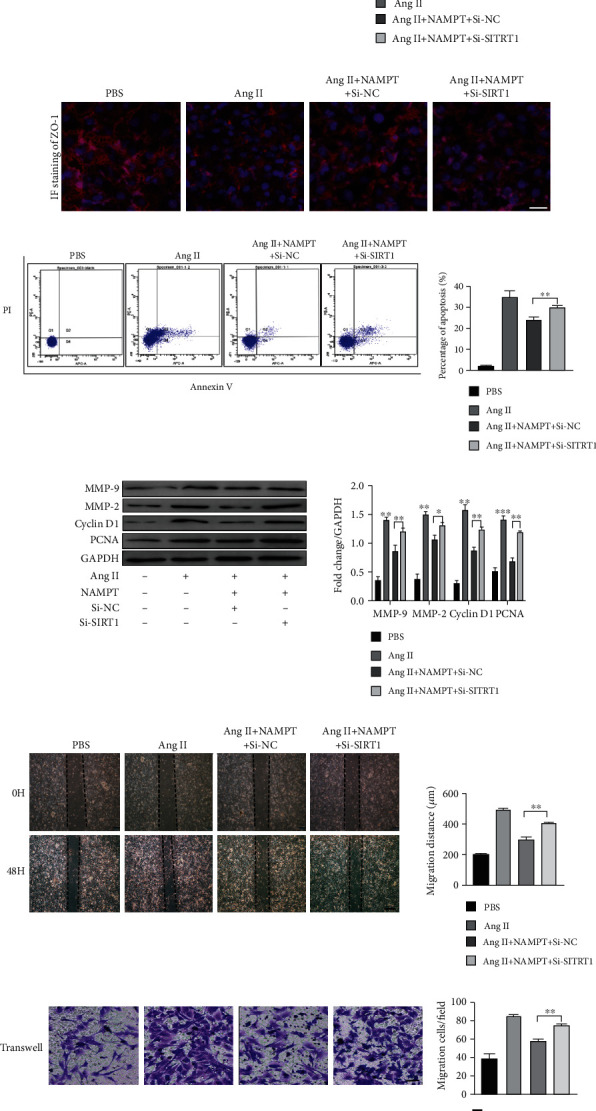
NAMPT alleviates Ang II-induced endothelial injury and changes in the proliferation and migration of MOVAs by targeting NAD/SIRT1 signaling. (a) The protein levels of apoptosis markers (Caspase 3 and Bcl-2) and P-eNOS/eNOS in NAMPT-overexpressing cells transfected with SIRT1-specific siRNA and stimulated with Ang II for 48 h were determined using Western blot analysis; the densitometric quantification is also shown. (b) The expression of the endothelial cell permeability marker ZO-1 in MAECs was determined using immunofluorescence staining (ZO-1: red; nuclei (DAPI): blue). Scale bar = 500 *μ*m. (c) MAECs subjected to different treatments were stained with Annexin V and PI and analyzed using flow cytometry to evaluate the effect of NAMPT on cell apoptosis after the Ang II treatment. Quantification of the cell apoptosis rate is shown in the right panel. (d) Relative levels of proliferation-related proteins (PCNA and cyclin D1) and extracellular matrix synthesis-related proteins (MMP-2 and MMP-9) in cultured MOVAs from the indicated groups. (e, f) Representative images of scratch-wound assays and transwell assays and quantification of migrated MOVAs (*n* = 4 in each group; scale bar = 200 *μ*m). Data are presented as means ± SEM.^∗^*p* < 0.05, ^∗∗^*p* < 0.01, and ^∗∗∗^*p* < 0.001 compared with the Ctrl or between the 2 indicated groups.

We designed a SIRT1-specific siRNA and transfected it into MAECs and MOVAs, followed by overexpression of NAMPT and Ang-II stimulation for 48 hours to further study the relationship between NAMPT and SIRT1. The expression of the SIRT1 mRNA was decreased in both cell lines (Fig. S2A and C). 48.26% decrease and 45.67% decrease in SIRT1 protein levels were observed in MAECs and MOVAs, respectively. Moreover, a 56.13% decrease and 58.11% decrease were observed after Ang II stimulation of these cells, respectively (Fig. S2B and D). Moreover, knockdown of SIRT1 rescued the expression of apoptosis markers (Caspase 3 and Bcl-2), which was also verified by PI/AnnexinV double staining and flow cytometry (Figure 5(c)). Similar results were obtained for the level of endothelial nitric oxide synthase (P-eNOS/eNOS) (Figure 5(a)). Immunofluorescence staining showed the restoration of the expression of the ZO-1 protein (an EC permeability marker) in SIRT1-silenced MAECs that overexpressed NAMPT and were stimulated with Ang II for 48 hours (Figure 5(b)). Moreover, the protein levels of proliferation markers (PCNA and cyclin D1) were also restored after SIRT1 silencing and NAMPT overexpression in cells stimulated with Ang II for 48 hours (Figure 5(d)). Scratch-wound and transwell migration assays revealed that cellular migration was also restored by SIRT1 knockdown (Figures 5(e) and 5(f)). The levels of the MMP-2 and MMP-9 mRNAs were also restored by silencing SIRT1 in MOVAs transfected with the NAMPT plasmid and stimulated with Ang II (Figure 5(d)). The intracellular and extracellular NAD levels were increased in MAECs and MOVAs transfected with the NAMPT plasmid and stimulated with Ang II (Fig. S3A-B).

### 2.6. NAMPT Reduced Intracellular ROS Levels through SIRT1 Signaling In Vivo and In Vitro

**Figure 6:**
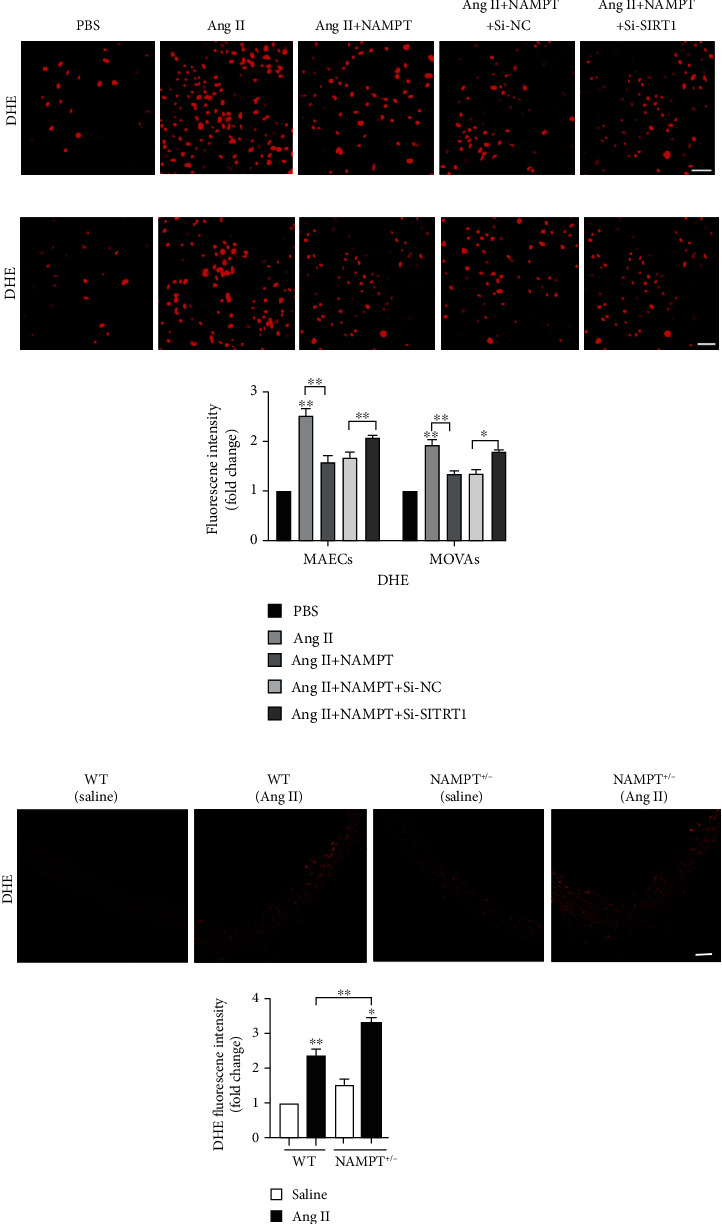
NAMPT reduces intracellular ROS levels through SIRT1 signaling in vivo and in vitro. DHE fluorescent probes and DCFH-DA were used to detect the level of ROS in MAECs (a) and MOVAs (b) with Sirt1 silencing, NAMPT overexpression, and Ang II stimulation. DHE (red) and DCF-DA (green). Scale bar = 500 *μ*m. (c) DHE staining of fresh aortic wall tissues from NAMPT+/- and WT stimulated with Ang II (*n* = 6 in each group). DHE (red). Scale bar = 50 *μ*m. Data are presented as means ± SEM. ^∗^*p* < 0.05 and ^∗∗^*p* < 0.01 compared with the corresponding saline group or between the 2 indicated groups.

According to previous studies, NAMPT mainly reduces oxidative stress. Dihydroethidium (DHE) fluorescent probes were used to detect the intracellular ROS levels and verify whether NAMPT alleviates oxidative stress induced by Ang II. After the overexpression of NAMPT and Ang II stimulation of MAECs for 48 hours, the ROS level was significantly reduced (Figure 6(a)). Moreover, the ROS level was significantly reduced by NAMPT overexpression in MOVAs stimulated with Ang II (Figure 6(b)). We subsequently knocked down SIRT1 using the SIRT1-specific siRNA to verify the effects of NAMPT/SIRT1 signaling. SIRT1 silencing restored the normal intracellular ROS level in MAECs (Figure 6(a)) and MOVAs (Figure 6(b)). Moreover, in vivo DHE staining showed an apparent increase in ROS levels in fresh aortic walls from NAMPT+/- mice compared with WT mice after Ang II stimulation (Figure 6(c)).

### 2.7. The MAPK Signaling Pathway Was Responsible for the Antihypertensive Effects of NAMPT/SIRT1

**Figure 7:**
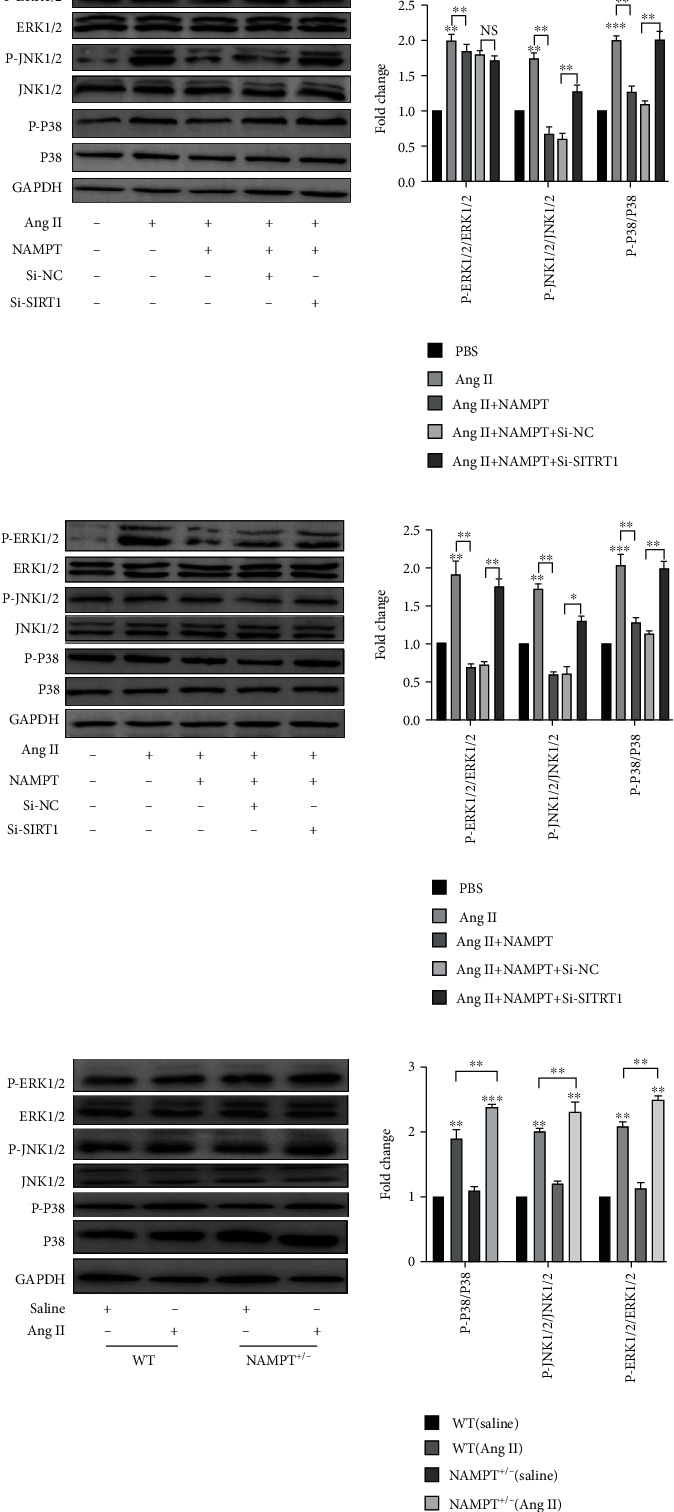
The MAPK signaling pathway is responsible for the antihypertensive effects of NAMPT/SIRT1. Western blots showing the levels of P-ERK1/2, P-JNK1/2, and P-P38 and quantification relative to the total ERK1/2, JNK1/2, and P38 levels in the MAECs (a) and MOVAs (b) transfected with the SIRT1-specific siRNA and NAMPT overexpression plasmid and stimulated with Ang II for 48 h. (c) Representative Western blots and quantitative analyses of the levels of the P-ERK1/2/ERK1/2, P-JNK1/2/JNK1/2, and P-P38/P38 proteins in fresh aortic wall tissues obtained from NAMPT+/- and WT mice after Ang II stimulation (*n* = 6 in each group). Data are presented as means ± SEM. ^∗^*p* < 0.05, ^∗∗^*p* < 0.01, ^∗∗∗^*p* < 0.001, and NS indicates no significant difference between the 2 indicated groups.

NAMPT has been shown to reduce oxidative stress caused by Ang II through SIRT1. Therefore, we examined the levels of the phosphorylated and total proteins for the kinases involved in the MAPK signaling pathway, specifically extracellular signal-regulated kinase 1/2 (ERK1/2), c-Jun N-terminal kinase 1/2 (JNK1/2), and P38, to investigate which downstream signaling pathways are modulated by ROS in vivo and in vitro. In MAECs, overexpression of NAMPT significantly reduced the levels of phosphorylated JNK and P38, but not ERK1/2. SIRT1 silencing rescued the levels of phosphorylated JNK1/2 and P38 in cells transfected with the NAMPT plasmid and stimulated with Ang II for 48 hours (Figure 7(a)). In MOVAs, the levels of phosphorylated ERK1/2, P38, and JNK1/2 were all decreased after transfection with NAMPT plasmids; moreover, SIRT1 silencing restored the levels of phosphorylated MAPK in cells transfected with the NAMPT plasmid and stimulated with Ang II for 48 hours (Figure 7(b)). We obtained fresh aortic walls from NAMPT+/- mice and WT mice after Ang II stimulation. The levels of phosphorylated MAPK pathway proteins, including ERK1/2, P38, and JNK1/2, were much higher in WT mice (Figure 7(c)).

### 2.8. Therapeutic Potential of rhNAMPT

**Figure 8:**
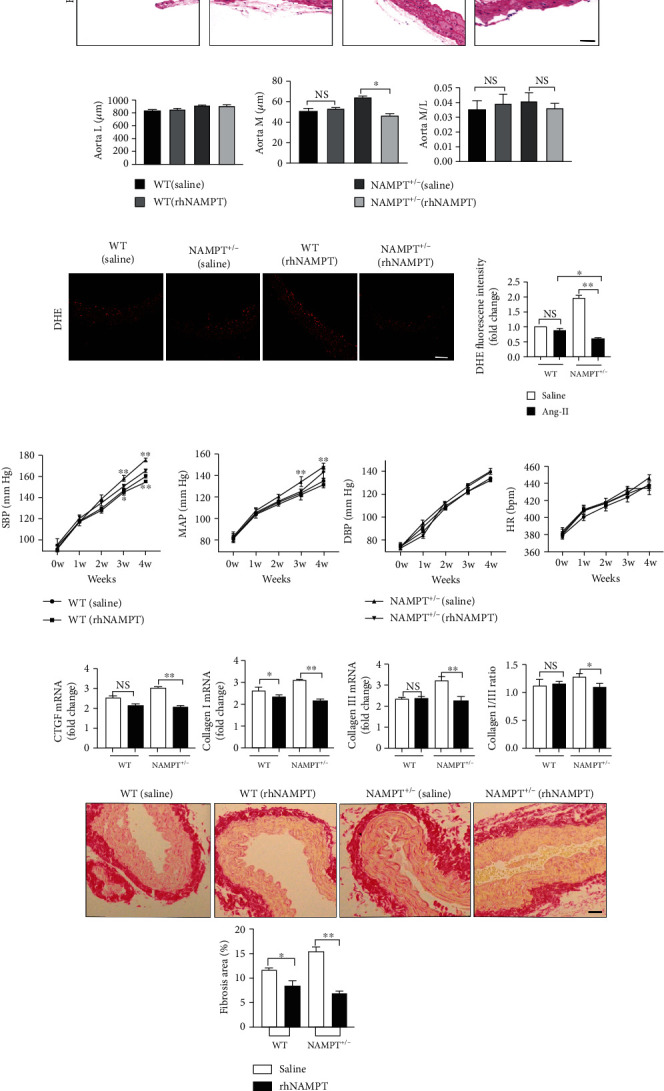
Therapeutic potential of rhNAMPT. (a) Representative images of H&E staining showing the remodeling of arteries (scale bar = 50 *μ*m, *n* = 5 in each group, ≥40 fields per group). (b) M, L, and their ratio in the aorta. (c) DHE staining of fresh aortic wall tissues from NAMPT+/- and WT mice after Ang II stimulation and rhNAMPT administration (DHE (red); *n* = 6 in each group, scale bar = 500 *μ*m). (d) The SBP, MAP, DBP, and HR were measured with a noninvasive computerized tail-cuff system in conscious mice. (e) Relative mRNA levels of CTGF, collagen I, and collagen III and the ratio of collagen type I and type III in WT littermates and NAMPT+/- mice treated with saline or Ang II treatment and injected with rhNAMPT (*n* = 6 in each group), as well as the picrosirius red staining (PSR). Data are presented as means ± SEM. ^∗^*p* < 0.05 and ^∗∗^*p* < 0.01 compared with the corresponding saline group; NS: not significant.

An intraperitoneal injection of rhNAMPT (2 *μ*g/100 g) was administered every 3 days from 1 week before surgically implanting the Ang II micropump to 4 weeks after surgery to investigate the therapeutic potential of rhNAMPT in vivo. The process continued until 4 weeks after surgery. H&E staining showed a slight decrease in the aortic M of NAMPT+/- mice after rhNAMPT administration, but the differences in L and M/L were not statistically significant in the different groups of mice during rhNAMPT treatment (Figures 8(a) and 8(b)). DHE staining was performed and suggested a significant alleviation of ROS levels in the aortic wall during rhNAMPT treatment compared with saline administration in NAMPT+/- and WT mice (Figure 7(b)). The ROS level in NAMPT+/- mice was obviously decreased compared with WT mice (Figure 8(c)). SBP, MBP, DBP, and HR were tested weekly to determine whether hypertension was ameliorated by rhNAMPT administration; a detailed description of blood pressure parameters is provided in Table S3. Ang II-induced changes in SBP and DBP were slightly alleviated by rhNAMPT administration to NAMPT+/- mice and WT mice. No significant differences in DBP or HR were observed (Figure 8(d)). Additionally, the qRT-PCR analysis revealed the downregulation of the Ang II-induced increase in the expression of remodeling markers, including CTGF, collagen I, and collagen III, in NAMPT+/- mice compared to WT mice (Figure 8(e)), as well as the picrosirius red staining (PSR). Moreover, NAD levels were also increased in plasma and aortic wall tissues from WT and NAMPT+/- mice treated with rhNAMPT (Fig. S3C).

## 3. Discussion

Based on our results, the expression of NAMPT was significantly downregulated in both patients with hypertension and experimental animals. In addition, NAMPT+/- mice exhibited significantly higher blood pressure and ROS levels than WT littermates following Ang II stimulation. Moreover, overexpression of NAMPT alleviated the Ang II-induced increase in ROS levels through SIRT1/NAD signaling in vivo and in vitro. Finally, the administration of rhNAMPT alleviated vascular remodeling in NAMPT+/- mice by modulating the oxidative stress response. Considering its antioxidant function, NAMPT may be a useful treatment for hypertension.

NAMPT was identified as an enzyme involved in NAD biosynthesis [7] and is also named pre-B cell colony-enhancing factor (PBEF) and visfatin [16]. NAMPT exerts both intracellular and extracellular effects on immunity, inflammation, metabolism, and stress responses under physiological and pathophysiological conditions [17, 18]. NAMPT modulates processes involved in the pathogenesis of obesity and related disorders by influencing the oxidative stress response [12], is often overexpressed in tumor tissues, and potentially contributes to the ability of cancer cells to resist oxidative stress [19]. The levels of NAMPT decrease with aging due to chronic inflammation, oxidative stress, and DNA damage [20]. In the cardiovascular system, NAMPT exacerbates atherosclerotic inflammation and promotes atherosclerosis development in ApoE mice [13]. NAMPT promotes pulmonary vascular remodeling, and its inhibition potentially represents a therapeutic strategy for pulmonary arterial hypertension (PAH) [14]. NAMPT inhibits myocardial injury in response to myocardial ischemia and reperfusion by regulating NAD and ATP levels [21]. Therefore, visfatin/NAMPT might be a novel therapeutic target in various disorders [22]. However, the molecular mechanism of NAMPT and its function in hypertension are not well understood. In the present study, the expression of NAMPT was significantly downregulated in both patients with hypertension and experimental animals. Moreover, the level of NAMPT was reduced in both MAECs and MOVAs after Ang II stimulation. Thus, we hypothesize that decreased NAMPT expression is associated with hypertension and may serve as a biomarker of hypertension.

As the initial effector molecule of the RAS, Ang II activation exerts an important effect on hypertension1 and has been shown to induce NOX activity and increase local ROS production by binding to AT1R [2]. ROS function as novel intracellular second messengers in signal transduction pathways that originate from a variety of membrane receptors. ROS promote endothelial dysfunction, which is an early event in the pathogenesis of hypertension [3]. Then, the proliferation and migration of VSMCs are critically involved in vascular remodeling processes [4]. These processes are also related to increased collagen decomposition, activation of MMPs, and ECM reorganization [23, 24]. In addition, the correlation between NAMPT-associated signaling and ROS has been detected in diverse diseases. NAMPT alleviates hypothalamic neuronal injury by reducing ROS production [25] and protects against ROS-induced inflammatory activation and DNA damage, which provides an integrated explanation for the dependence of inflammatory macrophages on the NAD salvage pathway [26]. NAMPT suppresses glucose deprivation-induced oxidative stress by increasing NADPH levels in breast cancer [27] and protects against myocardial injury in response to myocardial ischemia and reperfusion by regulating NAD+ and ATP levels [28]. Based on these findings, NAMPT plays an important role in ROS-associated injury [29]. However, the role of NAMPT in ROS-associated vascular remodeling remains unknown. Our study revealed a critical role for NAMPT in regulating Ang II-induced hypertension. NAMPT+/- mice exhibited significantly higher blood pressure and ROS levels than WT littermates after an Ang II infusion for 4 weeks. Moreover, the administration of rhNAMPT reduced the ROS level in NAMPT+/- mice, but the reduction in blood pressure was not obvious. NAMPT overexpression alleviated ROS-associated endothelial dysfunction, VSMC proliferation and migration, and ECM reorganization. Therefore, NAMPT may be employed in the clinic to prevent and potentially treat hypertension.

NAMPT modulates the activity of NAD-dependent enzymes, such as sirtuins [8], PARPs [9] and CD38 [10], thereby regulating cellular metabolism, mitochondrial biogenesis, and adaptive responses to inflammation, oxidation, and genotoxic stress [17, 18]. Sirtuins are a conserved family of enzymes present in diverse organisms. They possess NAD-dependent protein deacetylase activity, cleaving NAD during each deacetylation cycle [30]. Mammals express seven sirtuin homologs (SIRT1 to 7), of which SIRT1 is located in the nucleus and cytosol and, along with histone deacetylation, modulates transcription factors such as P53, nuclear factor kappa-light-chain-enhancer of activated B cells (NF-*κ*B), FOXOs, and PARP1. SIRT2 is a cytosolic sirtuin [31], while SIRT3, 4, and 5 are located in the mitochondria and have roles in oxidative stress and lipid metabolism. SIRT6 and 7 are nuclear sirtuins with roles in gene expression and DNA repair [30, 31]. We transfected both MOVAs and MAECs with NAMPT-overexpressing plasmids and stimulated them with Ang II to verify which sirtuins were involved. Interestingly, the expression of the SIRT1 mRNA was significantly increased compared to other SIRTs mRNAs in MOVAs and MAECs. The level of the SIRT1 protein was increased in vivo and in vitro. Moreover, NAMPT increased both extracellular and intracellular NAD concentrations. Importantly, the regulation of SIRT1 depended on NAMPT activity, which has been implicated in aging, metabolism, and tolerance to oxidative stress [32]. NAMPT promotes colorectal cancer cell growth via the SIRT1/P53 signaling pathway and is a prognostic marker in colorectal cancer [33]. The suppression of the NLRP3 inflammasome improves longevity and prevents cardiac aging in male mice through NAMPT/SIRT1 [34]. In cultured cardiomyocytes, NAMPT and SIRT1 cooperatively suppress the activity of mitochondrial proteins and ATP production, thereby promoting mitochondrial dysfunction [21]. Intracellular NAMPT has been recognized as the key enzyme involved in cellular NAD production. However, the biological functions of extracellular NAMPT are not clearly understood, and its receptor has not been identified. Researchers are still debating whether NAMPT has enzymatic activity outside the cell [11]. The intracellular NAMPT-NAD-SIRT1 cascade improves postischemic vascular repair by modulating Notch signaling in endothelial progenitors [35]. Meanwhile, extracellular NAMPT/visfatin causes P53 deacetylation via NAD production and SIRT1 activation in breast cancer cells [36]. Taken together, the role of the NAMPT/SIRT1 axis in the cardiovascular system remains controversial. In the present study, SIRT1 knockdown with a SIRT1-specific siRNA significantly exacerbated the Ang II-induced increases in the proliferation and migration of MOVAs and the apoptosis and endothelial nitric oxide synthase activity of MAECs after NAMPT overexpression, in addition to the ECM reorganization.

In turn, ROS activate a variety of protein tyrosine and serine/threonine kinases, many of which are known to promote multiple cellular responses, including growth, differentiation, apoptosis, and inflammation in diverse tissues and cells. Ang II-mediated ROS production activates the ERK signaling in ECs through the Ras/Raf/MEK pathway [37] and also induces JNK and P38-related signaling through apoptosis signaling kinase 1 (ASK1) [37, 38]. ROS promote the expression of vascular cell adhesion molecule-1 (VCAM1) and interstitial cell adhesion molecule-1 (ICAM1) and reduce intracellular nitric oxide synthase levels and eliminate endothelial NO, thereby decreasing EC viability [38, 39]. Ang II activates MAPK in VSMCs, promotes VSMC proliferation through ROS, and activates the expression of related proliferation genes such as AP-1, which further triggers the proliferation, migration, and secretion of ECM by VSMCs [40, 41]. Ang II activates JNK through c-Src and calcium ion-dependent protein kinase and promotes the proliferation of VSMCs [42]. In addition, c-Src activates ERK to promote migration. Furthermore, P38 MAPK plays an important role in activating heat shock protein 27- (HSP-27-) induced migration [40, 42], indicating that MAPK plays an important role in Ang II-mediated ROS-induced vascular remodeling. As shown in the present study, NAMPT overexpression alleviated the activation of ERK, P38 MAPK, and JNK induced by Ang II-mediated ROS production through SIRT1 in vivo and in vitro, but ERK activation in MAECs was not affected.

Collectively, this study is the first to show that NAMPT prevents Ang II-induced hypertension by inhibiting excess ROS accumulation, at least in part through the regulation of SIRT1 expression and the NAD concentration. Overall, NAMPT may be an innovative marker and therapeutic target for the intervention of hypertension and related vascular diseases.

The present study has several limitations. Firstly, due to laboratory conditions, we were unable to assess endothelium-dependent relaxation responses induced by acetylcholine (ACh) [43], which is a direct indicator of vascular endothelial function. Secondly, since NAMPT is expressed in almost all tissues in the body [11, 44], the effect of NAMPT knockout on the cardiovascular system is unknown. More experiments need to be performed, and conditional knockout mice should be created in sufficient numbers to obtain more powerful results in subsequent studies. Additionally, other signaling pathways may be associated with ROS, such as the PI3K/AKT, NF-*κ*B, and JAK/STAT pathways [45–47]. Further studies are needed to determine whether NAMPT regulates ROS activity through these signaling pathways.

## 4. Materials and Methods

### 4.1. Human Aortic Wall Tissue

All studies were approved by the local ethics committee of Tongji Hospital Affiliated to Tongji University and adhered to the principles of the Declaration of Helsinki. Aortic wall samples were obtained from the patients receiving aortic replacement surgery due to acute aortic dissection at Tongji Hospital from January 2018 to October 2019. Patients with chronic heart failure, chronic obstructive pulmonary disease, chronic kidney disease, and diabetes were excluded. Based on the presence or absence of hypertension, patients were divided into the hypertension group (*n* = 6) and the normal group (*n* = 6). No differences in the risk factors and medications were observed between the two groups (Table 1).

### 4.2. Animal Studies and Generation of NAMPT Haplodeficient Mice

Animal studies were approved by the Animal Care and Use Committee of Tongji Hospital Affiliated to Tongji University. The NAMPT haplodeficient mice (NAMPT+/-) were generated using CRISPR-Cas9 technology, as described previously [18]. The disruption of NAMPT by homologous recombination results in early embryonic lethality because of its dysfunction in egg cylinder organization and gastrulation. At the age of 8 weeks, male NAMPT+/- mice and their littermate wild-type (WT) mice were subcutaneously implanted with osmotic minipumps (model 2004; Alzet) to permit an infusion of Ang II (Sigma-Aldrich, Cat. no. A9525, 0.7 mg/kg per day) or saline for 4 weeks under inhalation anesthesia with 2% *v*/*v* isoflurane/oxygen, as described previously [44]. Blood pressure was measured weekly with a tail-cuff system using a heated scanner unit (BP-2010A, Softron) after surgery. The blood pressure of each mouse was detected at least five times until reaching a steady state, and the final value was calculated from an average of three proximal repeats.

### 4.3. Cell Culture and Treatment

MAECs and MOVAs were purchased from American Type Culture Collection (Manassas, VA, USA) and cultured in high-glucose DMEM supplemented with 10% fetal bovine serum and 1% penicillin/streptomycin. Moreover, MOVAs were incubated with 2 mmol/L L-glutamine. Cells were cultured in a 37°C, 5% CO_2_ incubator. Ang II was purchased from Sigma-Aldrich (Cat. no. A9525) and dissolved in sterile ultrapure water. Cells were exposed to Ang II at a final concentration of 10-6 mol/L and incubated for 24 or 48 hours.

### 4.4. Small Interfering RNA Transfection

The siRNA targeting mouse SIRT1 (5′-GCAGGTTGCAGGAATCCAAAG-3′) were synthesized by RiboBio Biotechnology Company (Guangzhou, China). For transfection, 50 nM SIRT1-siRNA and scrambled-siRNA were delivered into cells using the Lipofectamine™ 2000 transfection reagent (Invitrogen, Carlsbad, CA, USA) according to the manufacturer's instructions.

### 4.5. Transfection of pcDNA3.1-GFP-NAMPT Plasmids

After reaching 90% confluence, MOVAs and MAECs were transfected with 4 *μ*g of pcDNA3.1-GFP-NAMPT plasmids (Hanbio, Shanghai, China) using Lipofectamine™ 2000 (Invitrogen, Carlsbad, CA, USA) according to the manufacturer's instructions.

### 4.6. Quantitative Reverse Transcription PCR (qRT-PCR)

Total RNA was extracted from the aortic wall samples of rats, and MOVAs and MAECs were cultured using TRIzol (Takara). Total RNA (1ug) was reverse transcribed into cDNAs with the PrimeScript™ RT Reagent Kit (Takara). qRT-PCR was performed using SYBR Green (Takara), and the results were normalized to GAPDH expression. All primer sequences are shown in Table S1.

### 4.7. Western Blot Analysis

Proteins from the human aortic wall tissue of patients, aortic wall of mice, and cultured MOVAs and MAECs were extracted with RIPA lysis buffer (Thermo Fisher Scientific Inc.) supplemented with a protease and phosphatase inhibitor cocktail. Western blotting was performed using antibodies against NAMPT (Abcam, ab236874), P16 (Abcam, ab118459), P21 (Abcam, Ab109520), eNOS (Abcam, ab199956), P-eNOS (Abcam, ab32419), SIRT1 (Abcam, ab110304), MMP-2 (Abcam, ab97779), MMP-9 (Abcam, ab38898), AKT (CST, 4685S), P-AKT (CST, 4060S), P38 (CST, 8690S), P-P38 (CST, 4511S), ERK1/2 (Abcam, ab17942), P-ERK1/2 (Abcam, ab214362), JNK (Abcam, Ab179461), P-JNK (Abcam, Ab124956), and GAPDH (Abcam, ab8245), followed by an incubation with horseradish peroxidase-conjugated secondary antibodies (Santa Cruz Biotechnology) for 1 hour at room temperature. A gel imaging system (Tanon) and AlphaView software were used to image and analyze the Western blots.

### 4.8. Transwell Migration Assay and Scratch-Wound Assay

The transwell migration assay and scratch-wound assay were performed as previously described [26].

### 4.9. Histology, Immunohistochemical Staining, Immunofluorescence Staining, and Picrosirius Red Staining (PSR)

Aortic wall samples from the patients and mouse aortic wall tissues were fixed with 4% paraformaldehyde for 24 hours and then embedded in paraffin and sliced into 4 *μ*m sections. The morphology and fibrosis of the aortic wall were determined using H&E staining and picrosirius red staining (PSR) according to the manufacturer's instructions. Immunohistochemical staining was performed with antibodies against NAMPT (Abcam, ab236874) and SIRT1 (Abcam, ab110304). Immunofluorescence staining was performed using antibodies against CD31 (Abcam, ab28364), NAMPT (Abcam, ab236874), and ZO-1 (Abcam, ab190085) using previously described methods. Images were acquired and analyzed using Image-Pro Plus 6.0 software as previously described [25].

### 4.10. NAD Measurement

Intracellular and extracellular NAD levels were measured using an NAD/NADH analysis kit (Beyotime Biotechnology, Shanghai, China) according to the manufacturer's instructions. Briefly, cells or tissues were lysed, and the lysates were deproteinized with perchloric acid. Both the standard solution and samples were reacted with the developer in triplicate in a 96-well plate. After adding the specific enzyme, the plate was incubated at 37°C in the dark for 0.5 h. The absorbance of the resulting colored compound was measured at 450 nm.

### 4.11. Determination of ROS Levels

The MAECs, MOVAs, and fresh frozen vascular tissues were also stained with the DHE fluorescent probe (BestBio, Shanghai, China) diluted (1 : 1000 in cells and 1 : 500 in tissues) in serum-free medium to a final concentration of 5 *μ*M for cells and 10 *μ*M for tissues. Cells and tissues were incubated with the appropriate concentrations of DHE for 0.5 h at 37°C. The mean fluorescence intensity of DHE in the nuclei was measured. Finally, samples were detected using an excitation wavelength of 535 nm and an emission wavelength of 610 nm.

### 4.12. Data Analysis

Results are presented as means ± SEM. Two-tailed Student's tests were used for comparisons between two groups, and ANOVA followed by Tukey's post hoc test was used for comparisons of multiple groups. A *p* value less than 0.05 was considered statistically significant. GraphPad Prism 8.0 and SPSS 22.0 statistical software were used to analyze the data.

## Figures and Tables

**Table 1:** Clinical characteristics of patients with hypertension and normal undergoing aortic replacement.

	Normal group (*n* = 6)	Hypertension group (*n* = 6)	*p* value
Age (years, mean ± SD)	56.33 ± 7.68	68.25 ± 5.63	0.32
Male (*n*, %)	2 (33.3%)	3 (50%)	0.599
Systolic BP (mmHg, mean ± SD)	130.51 ± 4.38	174.50 + 8.65	0.002
Diastolic BP (mmHg, mean ± SD)	83.67 ± 5.39	101.17 + 9.46	0.008
Mean BP (mmHg, mean ± SD)	99.11 ± 4.12	125.44 + 8.64	0.006
Risk factors			
Smoking (*n*, %)	3 (50%)	2 (33.3%)	0.599
Hypercholesterolemia (*n*, %)	3 (50%)	4 (66.7)	0.609
Total cholesterol (mmol/L, mean ± SD)	5.09 ± 1.12	4.75 ± 0.84	0.507
BMI (kg/m^2^, mean ± SD)	25.97 ± 2.16	26.82 ± 3.05	0.493
LVEF (%)	55.33 ± 5.63	50.5 ± 8.35	0.344
Main medication			
Diuretics (*n*, %)	2 (33.3%)	3 (50%)	0.599
ACEI/ARB (*n*, %)	3 (50%)	4 (66.7)	0.609
*β* blockers (*n*, %)	5 (83.3%)	6 (100%)	0.341
Calcium antagonist (*n*, %)	2 (33.3%)	3 (50%)	0.599
Aldosterone antagonist (*n*, %)	3 (50%)	4 (66.7)	0.609
HMG CoA inhibitors (*n*, %)	2 (33.3%)	4 (66.7%)	0.29

BP: blood pressure; BMI: body mass index; ACEI: angiotensin-converting enzyme inhibitor; ARB: angiotensin receptor blocker; HMG-CoA: 3-hydroxy-3-methyl glutaryl coenzyme A.

## Data Availability

The data used to support the findings of this study are included within the article and the supplementary information file.

## Abbreviations

NAMPT: Nicotinamide phosphoribosyltransferase
SIRT1: Sirtuin 1
ROS: Reactive oxygen species
Ang II: Angiotensin II
MMP-2: Matrix metalloproteinase 2
MMP-9: Matrix metalloproteinase 9
ECM: Extracellular matrix
eNOS: Endothelial nitric oxide synthase
CTGF: Connective tissue growth factor
NAD: Nicotinamide adenine dinucleotide
MAECs: Mouse aortic endothelial cells
MOVAs: Mouse aortic vascular smooth muscle cells.

## Supplementary Materials

Supplementary 1 Figure S1: verification of overexpression plasmids in vitro. MAECs and MOVAs were transfected with NAMPT plasmids and then subjected to Ang II treatment for 48 h. Relative mRNA and protein expression of NAMPT was determined by qPCR and Western blot, respectively (A–D). The general structure of pcDNA3.1-GFP-NAMPT plasmids (E). Data were presented as mean ± SEM. ^∗^*p* < 0.05 and ^∗∗^*p* < 0.01 vs. the corresponding WT group or control.

Supplementary 2 Figure S2: verification of SIRT1-specific small interference RNA in vitro. MAECs and MOVAs were transfected with Si-SIRT1 and Si-NC, then subjected to Ang II treatment for 48 h. Relative mRNA and protein expression of SIRT1 was determined by qPCR (A)and Western blot (B) in MAECs, respectively, Moreover, relative mRNA and protein expression of SIRT1 was determined by qPCR (C) and Western blot (D) in MOVAs. Data were presented as mean ± SEM. ^∗∗^*p* < 0.01 and ^∗∗∗^*p* < 0.001 vs. control.

Supplementary 3 Figure S3: determination of NAD expression level in vivo and in vitro. Cells were transfected by sirt1-specific siRNA, followed by overexpression of NAMPT after Ang II stimulation for 48 hours; the intracellular and extracellular NAD levels of MAECs (A) and MOVAs (B). Representative NAD levels of plasma (C) and aortic wall from WT littermates and NAMPT+/- mice under saline or Ang-II treatment and rhNAMPT administration (*n* = 6 in each group). Data were presented as mean ± SEM. ^∗∗^*p* < 0.01 and ^∗∗∗^*p* < 0.001, and NS indicates no significance between the 2 indicated groups.

Supplementary 4 Table S1: the primer sequence for qRT-PCR.

Supplementary 5 Table S2: the SBP, MAP, and DBP of WT and NAMPT+/- mouse after Ang II infusion.

Supplementary 6 Table S3: the SBP, MAP, and DBP of WT and NAMPT+/- mouse after rhNAMPT and Ang II infusion.
