# Supplementary figures and images for: NAMPT/SIRT1 Attenuate Ang II-Induced Vascular Remodeling and Vulnerability to Hypertension by Inhibiting the ROS/MAPK Pathway
Source: Oxid Med Cell Longev. 2020 Dec 30;2020:1974265. doi: 10.1155/2020/1974265 (PMC7791967; doi:10.1155/2020/1974265)

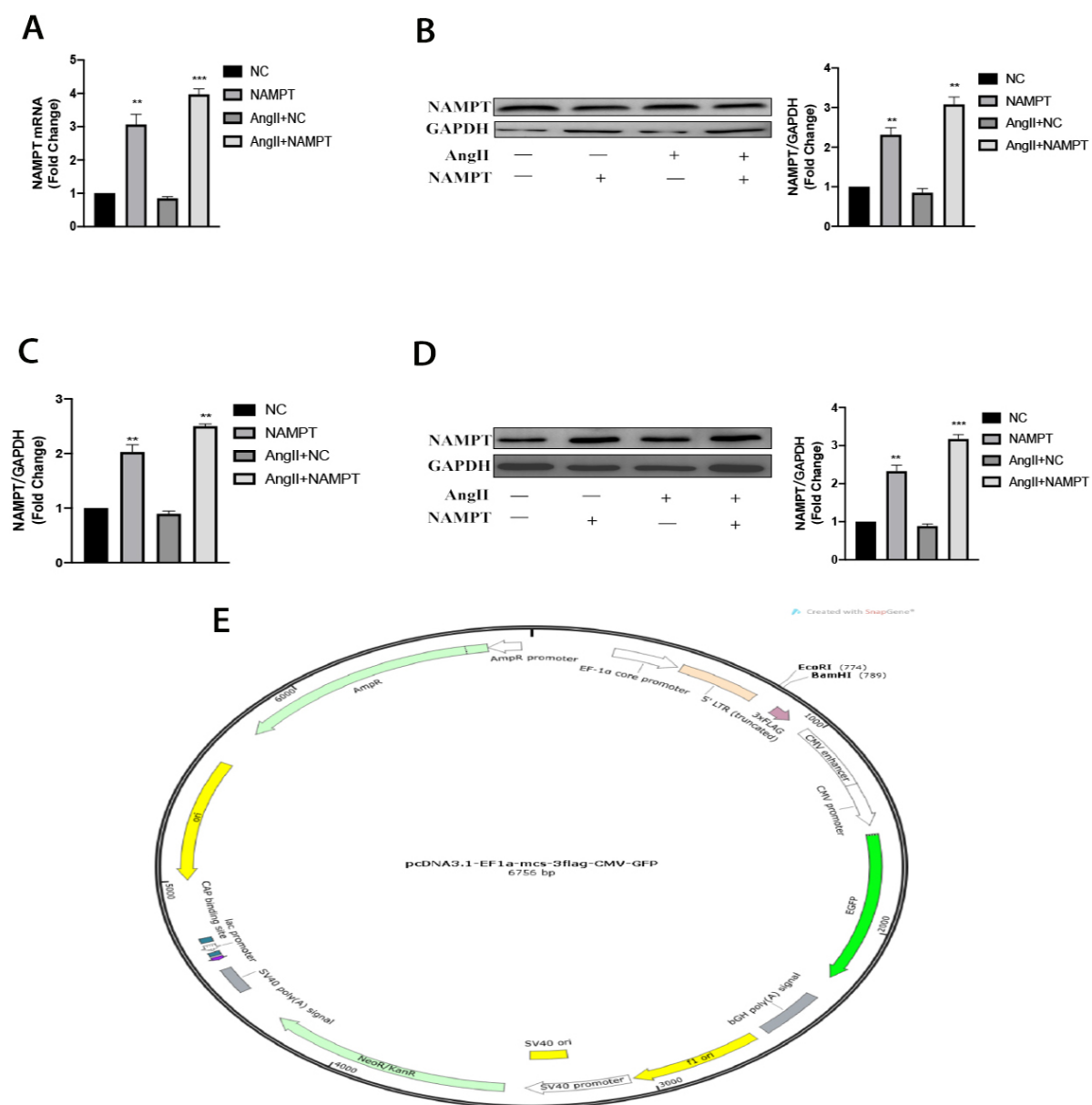

**Figure S1**

Supplement: Supplementary 1 — Figure S1: verification of overexpression plasmids in vitro. MAECs and MOVAs were transfected with NAMPT plasmids and then subjected to Ang II treatment for 48 h. Relative mRNA and protein expression of NAMPT was determined by qPCR and Western blot, respectively (A–D). The general structure of pcDNA3.1-GFP-NAMPT plasmids (E). Data were presented as mean ± SEM. ∗p < 0.05 and ∗∗p < 0.01 vs. the corresponding WT group or control. [file 1974265.f1.pdf]

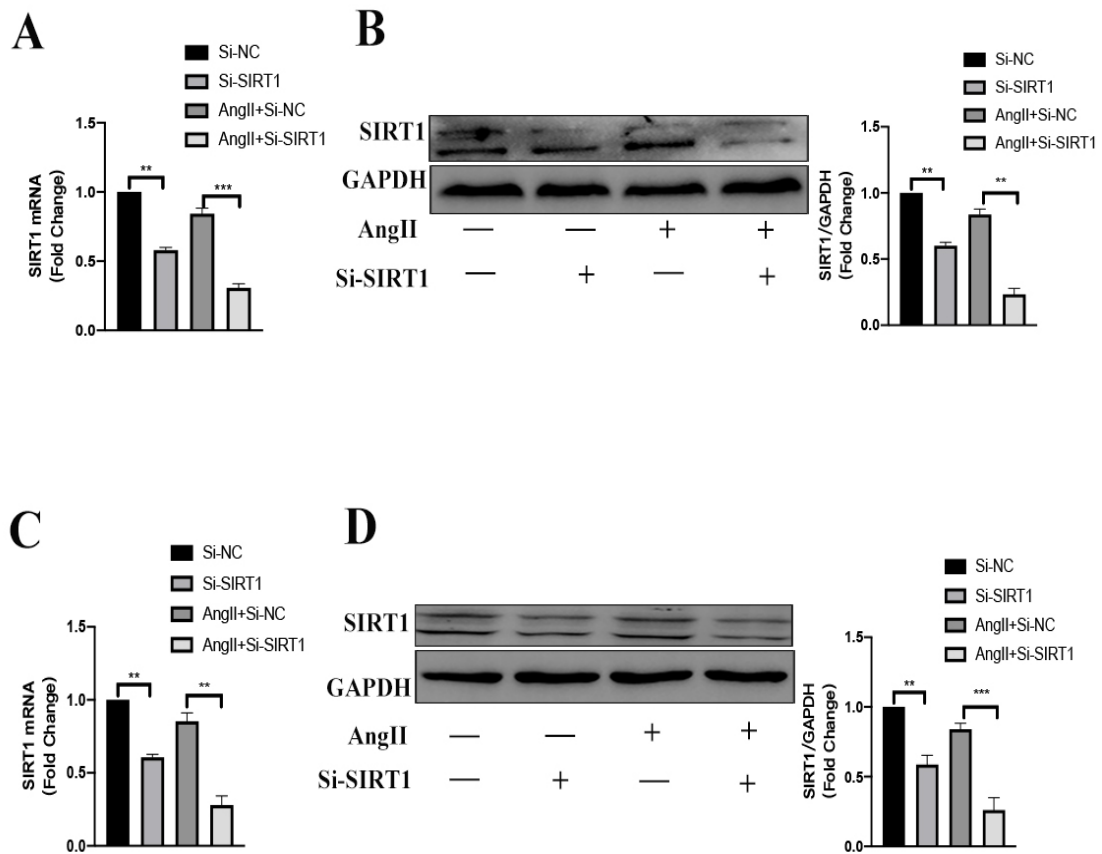

**Figure S2**

Supplement: Supplementary 2 — Figure S2: verification of SIRT1-specific small interference RNA in vitro. MAECs and MOVAs were transfected with Si-SIRT1 and Si-NC, then subjected to Ang II treatment for 48 h. Relative mRNA and protein expression of SIRT1 was determined by qPCR (A)and Western blot (B) in MAECs, respectively, Moreover, relative mRNA and protein expression of SIRT1 was determined by qPCR (C) and Western blot (D) in MOVAs. Data were presented as mean ± SEM. ∗∗p < 0.01 and ∗∗∗p < 0.001 vs. control. [file 1974265.f2.pdf]

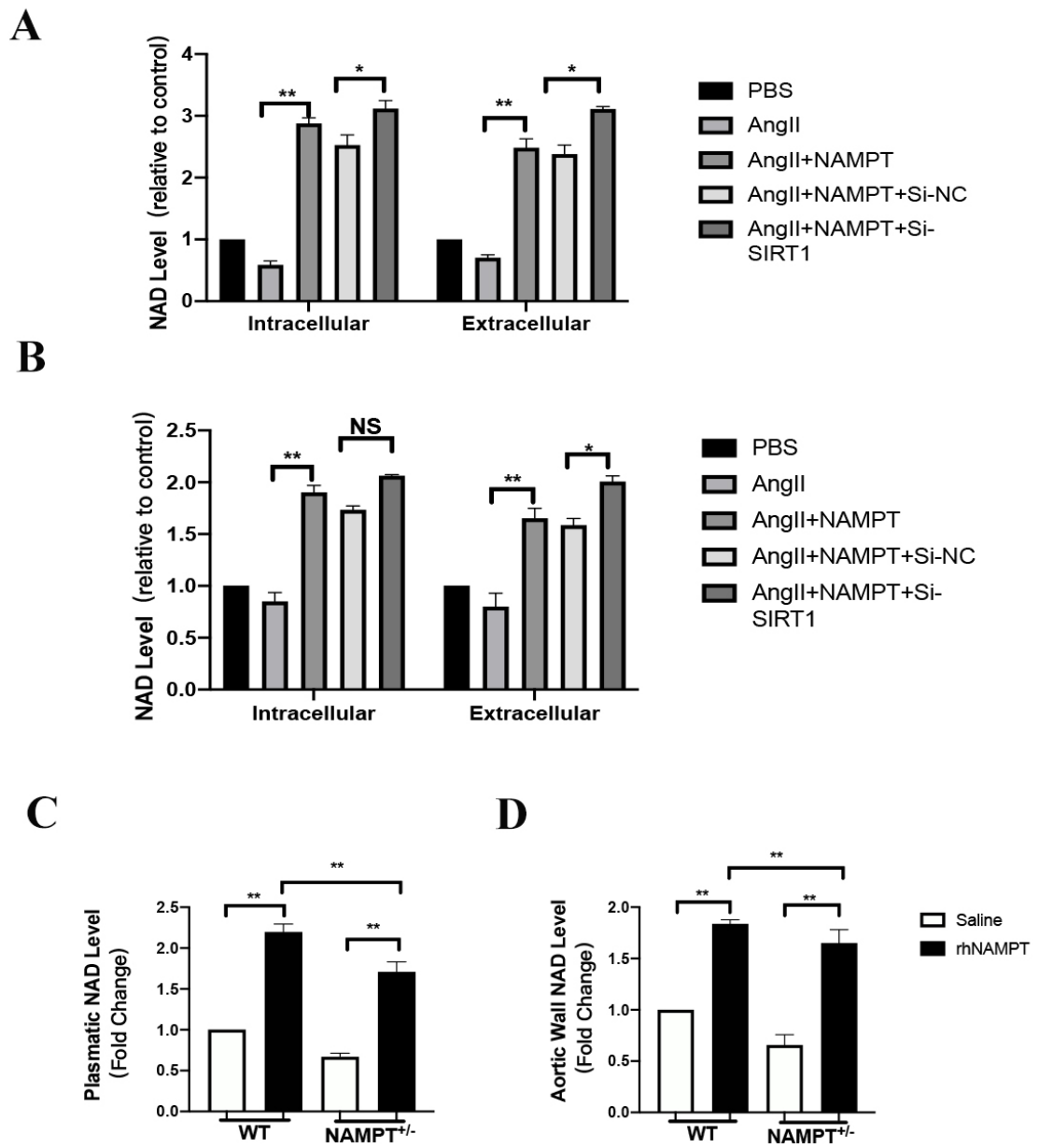

Figure S3

Supplement: Supplementary 3 — Figure S3: determination of NAD expression level in vivo and in vitro. Cells were transfected by sirt1-specific siRNA, followed by overexpression of NAMPT after Ang II stimulation for 48 hours; the intracellular and extracellular NAD levels of MAECs (A) and MOVAs (B). Representative NAD levels of plasma (C) and aortic wall from WT littermates and NAMPT+/- mice under saline or Ang-II treatment and rhNAMPT administration (n = 6 in each group). Data were presented as mean ± SEM. ∗∗p < 0.01 and ∗∗∗p < 0.001, and NS indicates no significance between the 2 indicated groups. [file 1974265.f3.pdf]
